# Complete blood count-derived inflammatory markers in canine cerebrovascular accidents: diagnostic utility and prognostic implications

**DOI:** 10.3389/fvets.2026.1719067

**Published:** 2026-04-22

**Authors:** Seungkuk Oh, SeJung An, Yeon-Jung Hong, DoHyeon Yu, Jinho Park

**Affiliations:** 1 College of Veterinary Medicine, Jeonbuk National University, Iksan, Republic of Korea; 2 School of Veterinary Medicine, University of Pennsylvania, Philadelphia, PA, United States; 3 College of Veterinary Medicine, Gyeongsang National University, Jinju, Republic of Korea; 4 Department of Veterinary Surgery, Western Referral Animal Medical Center, Seoul, Republic of Korea

**Keywords:** age-adjusted analysis, Canine Functional Stroke Score, cerebrovascular accident, neutrophil-to-lymphocyte ratio, prognostic factor, survival, systemic immune-inflammation index

## Abstract

**Background:**

Cerebrovascular accident (CVA) is an under-recognized cause of acute neurological dysfunction in dogs. Although magnetic resonance imaging (MRI) supports diagnosis, the limited access and overlapping imaging features complicate its interpretation. Blood-based inflammatory indices such as the neutrophil-to-lymphocyte ratio (NLR) and systemic immune-inflammation index (SII) have shown diagnostic and prognostic value in human stroke; however, their clinical relevance in canine CVA has not been validated.

**Objectives:**

To evaluate the diagnostic and prognostic utility of the NLR and SII in dogs with MRI-confirmed CVA compared to healthy controls.

**Methods:**

Seventeen dogs with CVA and 20 healthy controls were analyzed retrospectively. The NLR and SII were calculated from complete blood counts at presentation. Functional outcomes were assessed using the Canine Functional Stroke Score (CFSS₀, CFSS₃₀). Associations with 30-day survival were analyzed between groups.

**Results:**

Dogs with CVA had significantly higher NLR and SII values than healthy controls. The receiver operating characteristic curves yielded areas under the curves of 0.82 (95% CI: 0.68–0.96) for NLR and 0.85 (95% CI: 0.71–0.99) for SII. The optimal cutoffs determined by the Youden index were 3.92 for NLR (sensitivity 70.6%, specificity 90.0%) and 1,352 for SII (sensitivity 76.5%, specificity 90.0%). Both markers were moderately correlated with poorer 30-day recovery. The SII was significantly higher in non-survivors than in survivors. Baseline CFSS was not significantly correlated with the degree of functional improvement but was significantly higher in non-survivors. Multivariate Cox proportional hazards regression model, neither the NLR nor the SII were significantly associated with survival. Although individual predictors did not reach statistical significance, the overall model demonstrated a significant association with 30-day survival. In the dichotomized analysis, the SII model demonstrated an improved model fit for predicting mortality, whereas the NLR model showed a poorer fit. Kaplan–Meier analysis showed that the high-SII group had lower survival rates than the low-SII group, while no significant difference in survival was observed between the NLR groups.

**Conclusion:**

NLR and SII may help differentiate dogs with CVA from healthy dogs and reflect clinical outcomes. The SII also shows potential prognostic value for short-term survival.

## 1 Introduction

A cerebrovascular accident (CVA), commonly referred to as stroke, is defined as an episode of acute neurological dysfunction resulting from infarction or hemorrhage caused by cerebrovascular disease (1, 2). Although comprehensive prevalence data are unavailable, the reported incidence of CVA at a single referral hospital was estimated at 1.5–2% of neurological cases (3). The two primary subtypes of CVA are ischemic and hemorrhagic CVAs, which differ significantly in their frequency and pathophysiological characteristics (2). Ischemic CVA represents the most common form in dogs and results from vascular obstruction by emboli or local thrombus formation (3, 4). These CVAs are further classified as territorial if they affect a large artery or lacunar if they involve a small perforating artery (3). In contrast, hemorrhagic CVA arises from vessel rupture and is characterized by the extravasation of blood and formation of an intraparenchymal hematoma or diffuse infiltrate within the brain parenchyma (5). The diagnosis of CVA is frequently complicated by the lack of pathognomonic test results, limited accessibility to advanced imaging modalities, and overlapping magnetic resonance imaging (MRI) characteristics between CVA and neoplastic or inflammatory brain diseases (6–8). In addition to these diagnostic challenges, the assessment of prognosis and prediction of functional recovery in canine CVA remains difficult. To date, the absence of reliable biomarkers for assessing severity and predicting outcomes complicates clinical decision-making. Therefore, evaluating practical and accessible indicators that reflect the severity of the inflammatory response is necessary to improve prognostic assessment in affected dogs.

Both ischemic and hemorrhagic CVAs involve complex pathophysiological mechanisms, and inflammation plays a central role in their development and clinical progression (9–11). In particular, post-CVA inflammation has been identified as a major contributor to secondary brain injury (12). Consequently, various systemic inflammatory biomarkers have been explored in human medicine for their diagnostic and prognostic relevance in CVA (13).

The neutrophil-to-lymphocyte ratio (NLR), which is calculated by dividing the neutrophil count by the lymphocyte count, reflects both innate and adaptive immune responses and serves as a practical marker of systemic inflammation (14, 15). Elevated NLR values have been consistently associated with poor functional outcomes and increased mortality in patients with CVA (16). In veterinary medicine, the NLR has also been evaluated for its diagnostic and prognostic significance in conditions such as inflammatory bowel disease, meningoencephalitis of unknown origin (MUO), and lymphoma (17–19).

Another emerging biomarker is the systemic immune inflammation index (SII), which is calculated by multiplying the platelet count by the NLR (20, 21). Recent studies in humans have demonstrated that the SII is associated with CVA severity and prognosis and with post-CVA complications such as pneumonia and cognitive impairment (22–24). In veterinary medicine, the SII has been examined for its prognostic value under inflammatory conditions, including canine leishmaniasis and chronic inflammatory enteropathy (24, 25).

Despite growing evidence in the human literature, the diagnostic and prognostic significance of inflammatory markers such as the NLR and SII have not been clinically validated in dogs with cerebrovascular accidents. Therefore, this study aimed to evaluate the diagnostic and prognostic potential of the NLR and SII in dogs with MRI-confirmed cerebrovascular accidents. The hypothesis of this study was that the NLR can distinguish dogs with CVA from healthy controls and that both NLR and SII are associated with clinical outcomes following CVA.

## 2 Materials and methods

### 2.1 Study design

This study was a retrospective analysis of the medical records of client-owned dogs diagnosed with CVA at the Western Referral Animal Medical Center in Seoul, Korea, between June 2020 and March 2025. The collected data included age, breed, sex, neuter status, body weight, neurological signs, functional impairment assessed on admission, MRI findings, hematological parameters, and clinical outcomes.

### 2.2 Inclusion criteria

**Figure 1:**
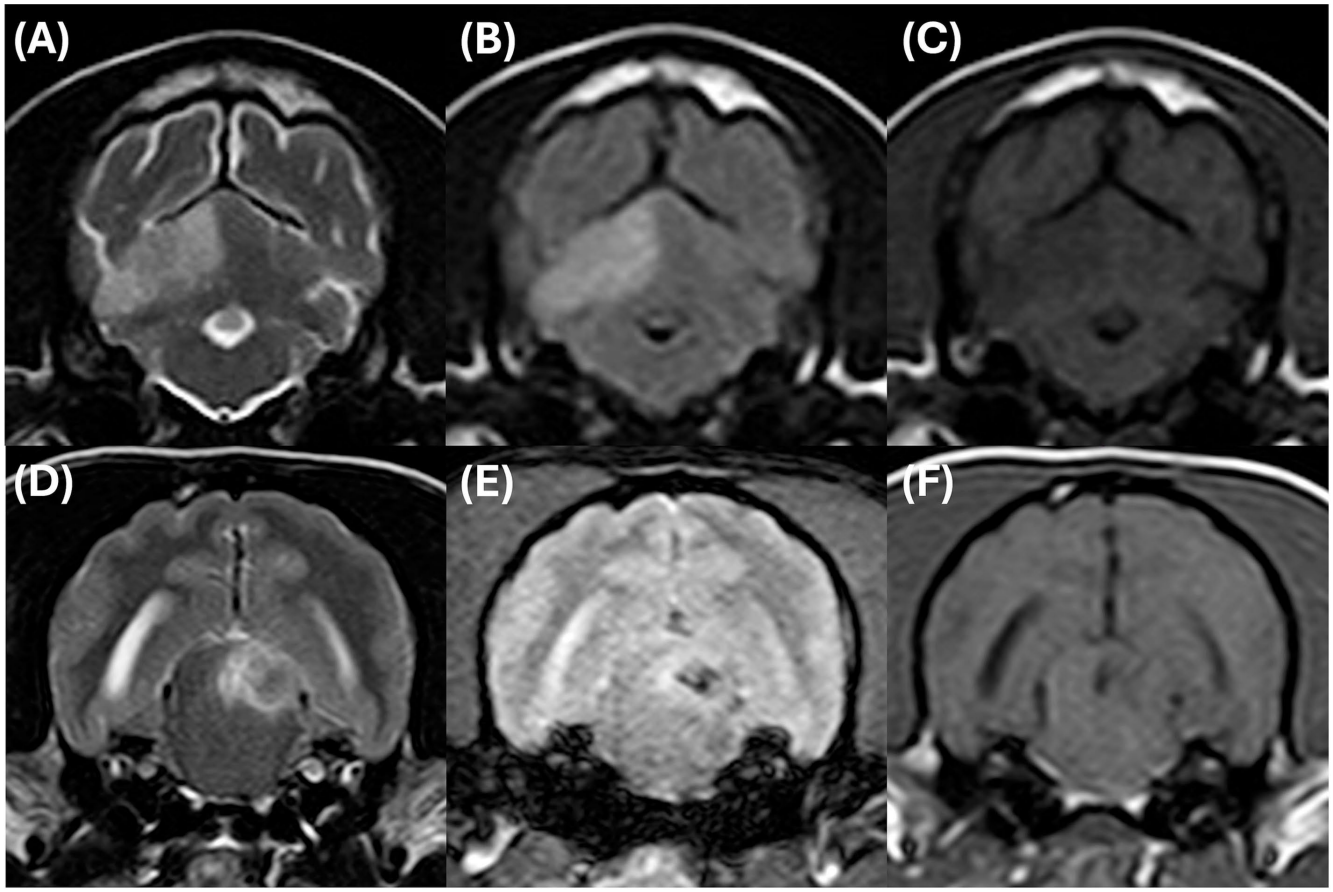
Representative magnetic resonance imaging (MRI) of ischemic and hemorrhagic cerebrovascular accidents (CVAs). Transverse images of an ischemic CVA (top row) show a well-demarcated, non-mass lesion in the rostral right cerebellar hemisphere, consistent with a vascular territory. The lesion appears hyperintense on T2-weighted **(A)** and FLAIR **(B)** images, hypointense on T1-weighted **(C)** images. Transverse images of a hemorrhagic CVA (bottom row) in the left midbrain reveal a mixed-intensity lesion on T2-weighted **(D)** images with prominent susceptibility effects on T2* **(E)** and a hypointense signal on T1-weighted **(F)** images.

The dogs included in this study were randomly selected client-owned dogs that presented with acute-onset neurological signs within 3 days of symptom onset and underwent MRI within 48 h of admission, confirming the diagnosis of CVA. MRI was performed within 48 h of admission using a 1.5 Tesla field strength scanner (Philips Achieva, Philips Medical Systems, Reigate, UK) to confirm the diagnosis of CVA. The imaging protocol included T1-weighted (pre- and post-contrast), T2-weighted, FLAIR, and T2* sequences. Post-contrast images were obtained following intravenous administration of gadopentetate dimeglumine (0.1 mmol/kg). Where available, diffusion-weighted imaging (DWI), susceptibility-weighted imaging (SWI), and apparent diffusion coefficient (ADC) maps were also reviewed. Due to the retrospective nature of the study, specific imaging parameters were adjusted based on clinical requirements. All images were independently reviewed by seven trained veterinary radiologists blinded to the clinical and hematologic data. An MRI-based diagnosis of CVA was made when findings were consistent with ischemic or hemorrhagic lesions and inconsistent with neoplastic or inflammatory disease, following established diagnostic criteria (Figure 1) (3, 6–8). Detailed descriptions of MRI findings and vascular territories for individual cases are provided in Supplementary Table S1.

Dogs were excluded if they had incomplete clinical data, were lost to follow-up, or had concurrent conditions that could have accounted for their neurological signs. Based on these criteria, 17 dogs were included in the final analysis. The healthy control group comprised 20 dogs retrospectively selected from those presented for routine health examinations. Controls were selected based on the absence of known medical conditions and no history of medications or treatments that could influence hematologic parameters or inflammatory status.

### 2.3 Neurological assessment and clinical outcome evaluation

Neurological functional status was assessed using the Canine Functional Stroke Score (CFSS), a six-grade ordinal scale adapted from human stroke scoring systems for application in dogs. The CFSS is conceptually derived from the modified Rankin Scale (mRS), which assesses overall functional independence, and from the National Institutes of Health Stroke Scale (NIHSS), which quantifies neurological deficits across multiple domains in human patients (26, 27). The CFSS was designed to incorporate key functional parameters observable in dogs, including ambulation, posture, mentation, and the presence and severity of neurological deficits, such as ataxia, paresis, and postural reaction deficits. The scores range from 0 (normal function) to 5 (coma or death). The full definitions of each CFSS grade are provided in Table 1.

**Table 1:** Canine Functional Stroke Score (CFSS) (24, 25).

Score	Functional level	Description
0	Normal	Normal ambulation, posture, and mentation. No neurological deficits
1	Mild deficit	Independent ambulation with mild ataxia or proprioceptive deficits. Neurological abnormalities are subtle and do not interfere with daily life
2	Moderate deficit	Ambulatory with clear ataxia or paresis and obvious postural reaction deficits. May require minimal assistance
3	Moderate-to-severe deficit	Non-ambulatory but able to maintain sternal posture. Severe ataxia, tetraparesis, or marked postural reaction deficits. Conscious and responsive
4	Severe deficit	Nearly no voluntary movement. Tetraplegia. Severe impairment of posture and mentation. Pain perception present, but mentation depressed
5	Coma or death	No response to pain, absent voluntary movement, comatose, or deceased. Complete neurological failure

The CFSS was adapted from the modified Rankin Scale and the National Institutes of Health Stroke Scale, both of which are widely used for functional assessment in patients with cerebrovascular accident.

Neurological assessments were performed at two standardized timepoints: Day 0 (initial presentation; CFSS₀) and Day 30 (CFSS₃₀). Neurological assessments were performed by the attending clinicians responsible for each case. While scoring was based on standardized clinical evaluation protocols, assessments were conducted as part of routine clinical care without separate inter-rater calibration. The CFSS values were retrospectively extracted from the medical records for analysis. Clinical improvement was defined as a reduction in the CFSS over time, with greater decreases indicating more substantial recovery. For statistical analysis, changes in CFSS between timepoints (ΔCFSS₀_‒_₃₀) were calculated and analyzed descriptively.

### 2.4 Hematological and inflammatory marker analysis

Peripheral blood samples were analyzed using an ADVIA 2120i automated hematology analyzer (Siemens Healthineers, Erlangen, Germany) to obtain complete blood count (CBC) parameters. The analyzer was subjected to daily quality control and calibration according to the manufacturer’s guidelines. Due to the retrospective nature of the study, fasting status at the time of blood collection was not standardized. All samples were analyzed within 1 h of collection to ensure data integrity. Blood samples for hematological analysis were collected in ethylenediaminetetraacetic acid tubes.

The NLR was calculated by dividing the absolute neutrophil count by the absolute lymphocyte count (14). The SII was calculated by multiplying the platelet count by the NLR (20). WBC parameters were analyzed without excluding potential stress-related changes to reflect the overall clinicopathological status at presentation.

### 2.5 Statistical analysis

Statistical analyses and figure generation were primarily performed using GraphPad Prism version 10.5 (GraphPad Software, San Diego, CA, United States). Cox proportional hazards regression analysis, verification of the proportional hazards assumption, and internal validation of the Receiver operating characteristic (ROC) analysis were conducted using R version 4.5.2 (R Foundation for Statistical Computing, Vienna, Austria).

The Shapiro–Wilk test was used to assess normality. Comparisons between dogs with CVA and healthy controls were performed using the Mann–Whitney U test for continuous variables, including age, body weight, and all hematologic and inflammatory parameters. Within the CVA group, the Mann–Whitney U test was also used to compare CFSS₀ between 30-day survivors and non-survivors. Sex distribution between groups was compared using Fisher’s exact test. To account for multiple comparisons across all clinical and hematological parameters, the Benjamini-Hochberg procedure was applied to control the false discovery rate (FDR). Results were reported as *q*-values, and a *q*-value of less than 0.05 was considered statistically significant. Additionally, to evaluate the magnitude of the difference between groups, the standardized effect size (*r*) was calculated using the rank-biserial correlation, defined as *r* = 1 − (2 U / (*n*_1_ × *n*_2_)). The effect size was interpreted according to Cohen’s guidelines: 0.1 ≤ |*r*| < 0.3 as a small effect, 0.3 ≤ |*r*| < 0.5 as a medium effect, and |*r*| ≥ 0.5 as a large effect (28).

ROC curve analysis was used to evaluate the diagnostic performance of the NLR and SII in differentiating dogs with CVA from healthy controls. The area under the curve (AUC) was calculated, and diagnostic accuracy was categorized as sufficient (AUC 0.6–0.7), good (0.7–0.8), very good (0.8–0.9), or excellent (0.9–1.0). The optimal cut-off value for each marker was determined using the Youden index (sensitivity + specificity − 1), and the corresponding sensitivity and specificity were reported (29, 30). To evaluate the stability of these thresholds and associated diagnostic parameters, including sensitivity, specificity, positive predictive value (PPV), and negative predictive value (NPV), internal validation was performed using bootstrapping with 2,000 replicates. All bootstrapped 95% CIs were calculated using the pROC package in R software (31). To assess the independent diagnostic value of each marker while adjusting for age, multivariable logistic regression was performed to estimate age-adjusted AUCs.

Spearman’s rank correlation analysis was performed to examine associations between inflammatory markers (NLR and SII) and clinical parameters, including the CFSS₀, ΔCFSS₀_‒_₃₀, and the relationship between CFSS₀ and ΔCFSS₀_‒_₃₀. For dogs that died before the 30-day evaluation, a CFSS score of 5 was assigned to day 30. Correlation coefficients (*r*) were interpreted as negligible (|*r*| < 0.3), low (0.3 ≤ |*r*| < 0.5), moderate (0.5 ≤ |*r*| < 0.7), or strong (|*r*| ≥ 0.7), and *p* < 0.05 was considered statistically significant (32). A sensitivity analysis was also performed by excluding euthanized cases to verify the robustness of the correlation between inflammatory markers and ΔCFSS₀_‒_₃₀.

Cox proportional hazards regression analysis was used to evaluate the association between the inflammatory markers and 30-day survival. Hazard ratios (HR) and 95% confidence intervals (CI) were reported, and the model fit was assessed using Akaike’s Information Criterion (AIC) (33, 34). For the survival analysis, dogs that died or were euthanized due to severe and persistent neurological deficits attributed to the cerebrovascular accident were classified as events. The survival time was defined as the number of days from the onset to death or CVA-related euthanasia, with follow-up administratively censored at 30 days. Dogs that were alive at day 30 were right-censored. To ensure clinical interpretability, the SII was analyzed as a scaled continuous variable (per 1,000-unit increase). The proportional hazards assumption was verified using Schoenfeld residual tests (35).

To address potential non-linear effects and enhance clinical interpretability, the NLR and SII were dichotomized based on their optimal cutoff values. Dichotomized Cox regression was performed to assess the association between high- and low-marker groups and 30-day survival. Kaplan–Meier survival curves were generated for these groups, and differences in survival distributions were evaluated using the log-rank test.

For all analyses, two-tailed exact *p* values were calculated, and *p* values < 0.05 were considered statistically significant.

All data are presented as medians and interquartile ranges.

## 3 Results

### 3.1 Study population

The healthy dogs included four Bichon Frises, four Maltese, three Poodles, two Yorkshire Terriers, and one each of the American Cocker Spaniel, Welsh Corgi, American Bulldog, mixed-breed dog, Pomeranian, Spitz, and Dachshund.

The CVA group included five Maltese, three Poodles, two mixed-breed dogs, two Pomeranians, two Spitz, and one each of Yorkshire Terrier, Dachshund, and Chihuahua. The demographic characteristics of the two groups are summarized in Table 2. Although dogs with CVA were significantly older than healthy dogs (*p* = 0.003), there were no statistically significant differences in body weight or sex distribution between the two groups.

**Table 2:** Characteristics of healthy dogs and dogs with CVA.

Variable	Healthy (*n* = 20)	CVA (*n* = 17)	*p* value	*q* value	Effect size (*r*)
Age (years)	9.0 (6.7–10.8)	13.3 (10.0–15.0)	0.003**	0.003^†^	0.562
Body weight (kg)	6.9 (3.7–9.1)	4.3 (2.9–6.1)	0.195	0.130	0.253
Sex (*n*, %)			0.331		
Male	7 (35.0%)	9 (52.9%)			
Female	13 (65.0%)	8 (47.1%)			

Age and body weight are expressed as medians and interquartile ranges and were compared between groups using the Mann–Whitney U test. False discovery rate was used for multiple comparison correction. Effect size was calculated for each comparison. The values for sex are expressed as the number of dogs (percentage of total instances) and were compared using Fisher’s exact test. Dogs with CVA were significantly older than healthy dogs (*p* = 0.003). CVA, Cerebrovascular accident. *p < 0.05; **p < 0.01; ***p < 0.001. ^†^*q* < 0.05.

### 3.2 Hematologic variables and CFSS scores

The results of hematological analyses of healthy dogs and dogs with CVA are summarized in Table 3. Dogs with CVA exhibited significantly lower hematocrit, red blood cell count, and hemoglobin concentration compared to healthy dogs, all demonstrating large effect sizes. In contrast, the CVA group showed significantly higher white blood cell and monocyte counts with large effect sizes. The platelet count was found to be statistically significant after FDR correction with a medium effect size. No other hematological variables showed significant differences between groups. The median CFSS score in dogs with CVA decreased from 3.0 (3.0–4.0) at admission to 2.0 (1.0–5.0) at day 30.

**Table 3:** Variables in healthy dogs and dogs with CVA.

Variable	Healthy (*n* = 20)	CVA (*n* = 17)	Reference interval	*p* value	*q* value	Effect size (*r*)
HCT (%)	53.30 (49.63–55.00)	46.30 (40.69–48.40)	37.1–57.0	0.001**	0.002^†^	0.606
RBC (10^6^/μL)	7.70 (7.35–8.48)	6.63 (6.19–7.46)	5.7–8.8	0.007**	0.005^†^	0.518
Hgb (g/dL)	17.55 (16.10–18.40)	15.30 (13.80–16.50)	12.9–18.4	0.004**	0.004^†^	0.538
MCV (fL)	67.80 (65.70–70.20)	67.40 (65.20–72.70)	58.8–71.2	0.839	0.411	0.041
MCHC (g/dL)	33.10 (32.20–33.35)	33.10 (31.40–33.90)	31.0–36.2	0.982	0.451	0.006
PLT (10^3^/μL)	323.50 (294.00–414.75)	424.00 (325.00–489.00)	143.3–400	0.064	0.047^†^	0.359
WBC (10^3^/μL)	8.27 (7.04–9.45)	11.04 (9.59–17.99)	5.2–13.9	<0.001***	0.001^†^	0.632
Neutrophil (10^3^/μL)	5.52 (3.78–6.03)	7.90 (7.30–14.69)	3.9–8.0	*<*0.001***	<0.001^†^	0.691
Lymphocyte (10^3^/μL)	2.30 (2.00–2.61)	1.83 (1.38–2.80)	1.3–4.1	0.446	0.234	0.150
Monocyte (10^3^/μL)	0.42 (0.32–0.49)	0.75 (0.50–0.80)	0.2–1.1	0.001***	<0.001^†^	0.656
Eosinophil (10^3^/μL)	0.29 (0.19–0.43)	0.28 (0.10–0.44)	0–0.6	0.354	0.217	0.335
Basophil (10^3^/μL)	0.01 (0.01–0.01)	0.01 (0.00–0.02)	0–0.1	0.422	0.234	0.306

Data are presented as medians and interquartile ranges and were compared between groups using the Mann–Whitney U test. False discovery rate was used for multiple comparison correction. Effect size was calculated for each comparison. CVA, Cerebrovascular accident; HCT, Hematocrit; Hgb, Hemoglobin; MCV, Mean corpuscular volume; MCHC, Mean corpuscular hemoglobin concentration; PLT, Platelet; RBC, Red blood cell; WBC, White blood cell. *p < 0.05; **p < 0.01; ***p < 0.001. ^†^*q* < 0.05.

### 3.3 Association between initial neurologic status and clinical outcome

**Figure 2:**
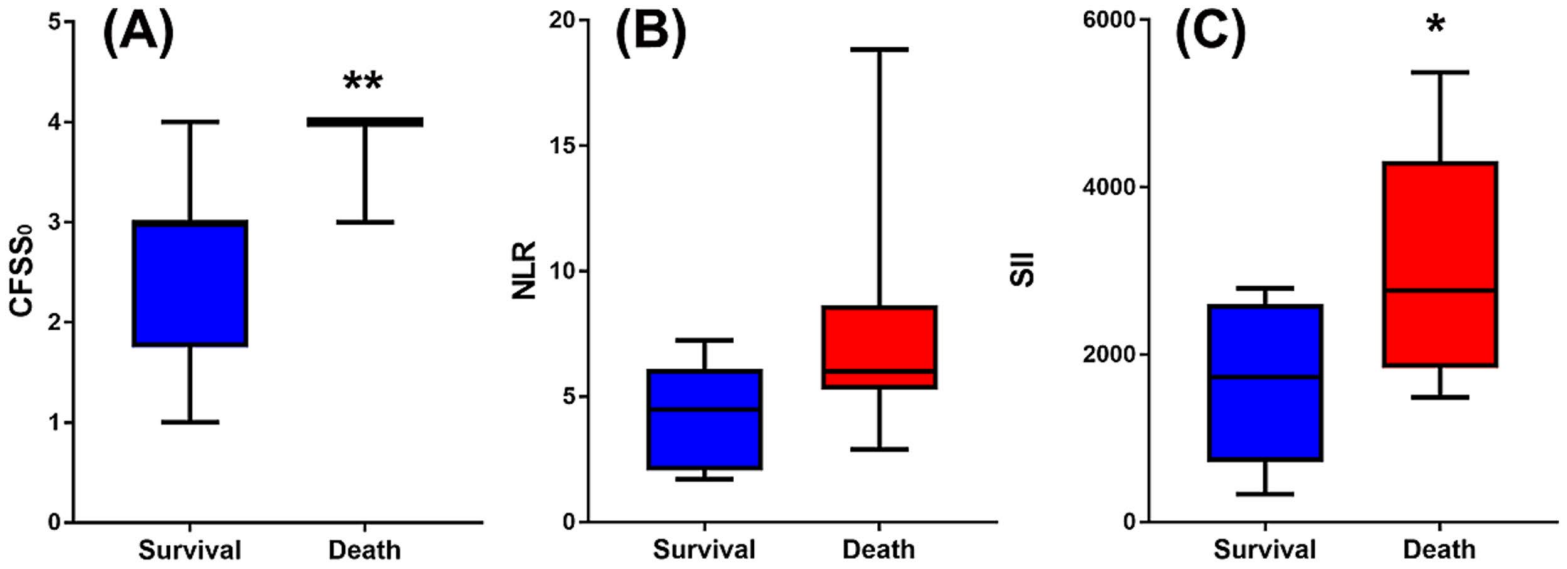
Data comparison between 30-day survival and non-survival. **(A)** CFSS₀, **(B)** the NLR, and **(C)** the SII at presentation between 30-day survivors and non-survivors among dogs with CVA. CFSS₀ was significantly higher in non-survivors than in survivors **(A)**. NLR was higher in non-survivors than in survivors, but the difference was not statistically significant **(B)**. SII was significantly elevated in non-survivors compared to that in survivors **(C)**. Horizontal bars indicate medians with interquartile ranges. Statistical significance was determined using the Mann–Whitney U test. *p < 0.05; **p < 0.01. CFSS₀, Canine Functional Stroke Score at initial presentation; CVA, cerebrovascular accident; NLR, neutrophil-to-lymphocyte ratio; SII, systemic immune-inflammation index.

To evaluate whether the baseline neurological status was associated with the degree of functional improvement, a correlation analysis was performed between CFSS₀ and ΔCFSS₀_‒_₃₀. This analysis revealed a negative but non-significant association (*r* = −0.30; 95% CI: −0.69 to 0.22; *p* = 0.235), indicating that initial functional severity did not predict the magnitude of recovery. In contrast, CFSS₀ scores were significantly higher in dogs that died within 30 days than in survivors (*p* < 0.001), suggesting that more severe initial neurological deficits were associated with short-term mortality (Figure 2).

### 3.4 Comparison of inflammatory markers between healthy dogs and dogs with CVA

**Figure 3:**
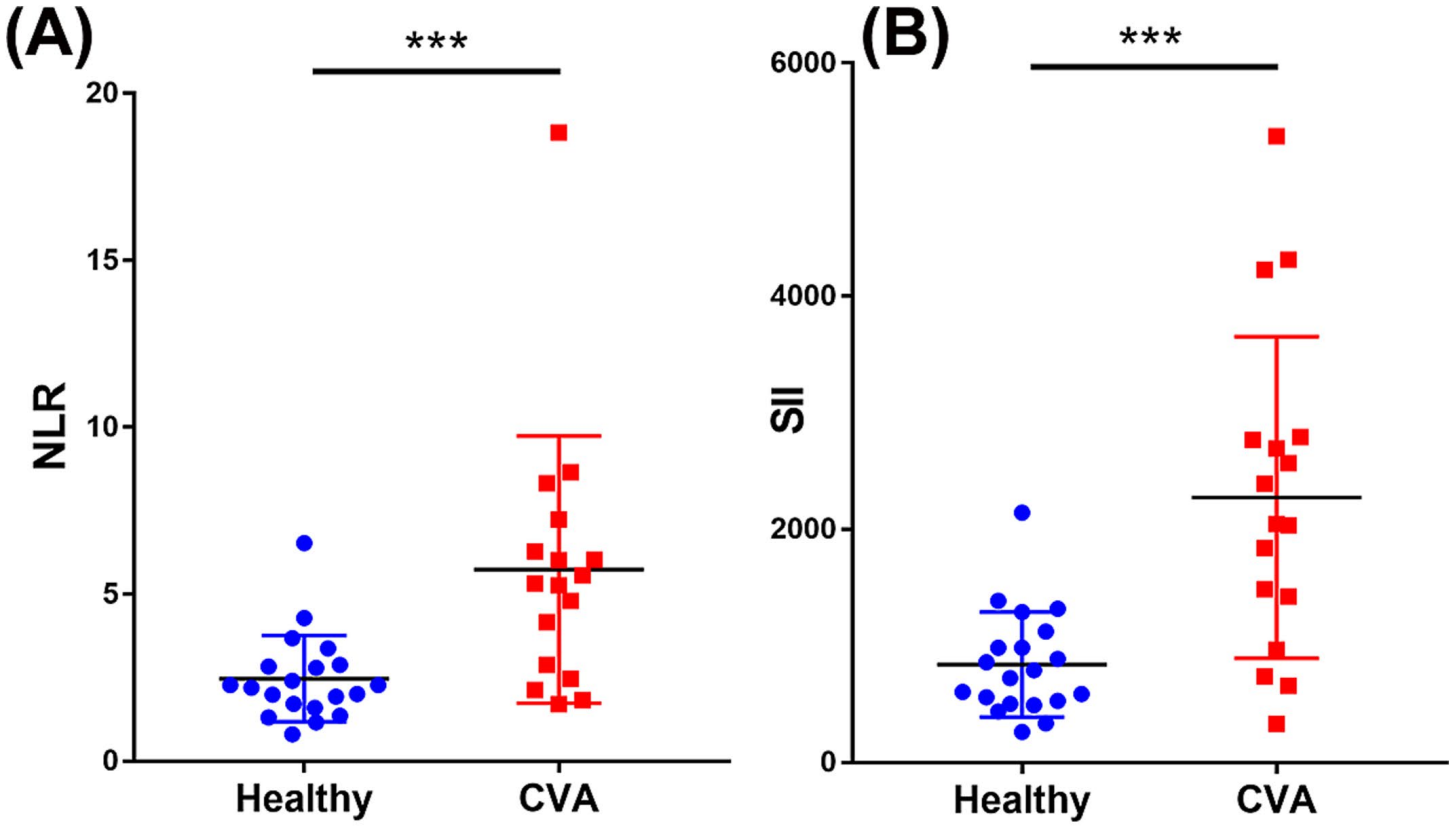
Scatterplots comparing the neutrophil-to-lymphocyte ratio (NLR) **(A)** and systemic immune-inflammation index (SII) **(B)** between dogs with cerebrovascular accident (CVA; *n* = 17) and healthy controls (*n* = 20). **(A)** NLR was significantly higher in dogs with CVA than in healthy dogs. **(B)** SII was significantly higher in the CVA group than in healthy controls. Horizontal bars indicate medians with interquartile ranges. Statistical significance was determined using the Mann–Whitney *U* test. ***p < 0.001. CVA, cerebrovascular accident; NLR, neutrophil-to-lymphocyte ratio; SII, systemic immune-inflammation index.

Both the NLR and SII were significantly higher in dogs with CVA than in healthy dogs exhibiting large effect sizes. The NLR was 5.33 [2.88–6.29] in the CVA group and 2.24 [1.69–2.85] in the healthy group (*p* < 0.001; *q* < 0.001; *r* = 0.644). The SII was 2045.39 [1424.57–2767.02] in the CVA group and 757.83 [523.17–1019.55] in the healthy group (*p* < 0.001; *q* < 0.001; *r* = 0.694) (Figure 3).

### 3.5 Diagnostic performance of inflammatory markers in CVA

The ROC analysis demonstrated that both the NLR and SII exhibited good diagnostic performance in differentiating dogs with CVA from healthy controls. For the NLR, the AUC was 0.82 (95% CI: 0.68–0.96; *p* < 0.001), and the optimal cutoff value was 3.92. At this threshold, the sensitivity and specificity were 70.6% (95% CI: 47.1–88.2%) and 90.0% (95% CI: 75.0–100.0%), respectively. The PPV was 86.2% (95% CI: 68.8–100.0%) and the NPV was 78.3% (95% CI: 66.7–90.9%).

**Figure 4:**
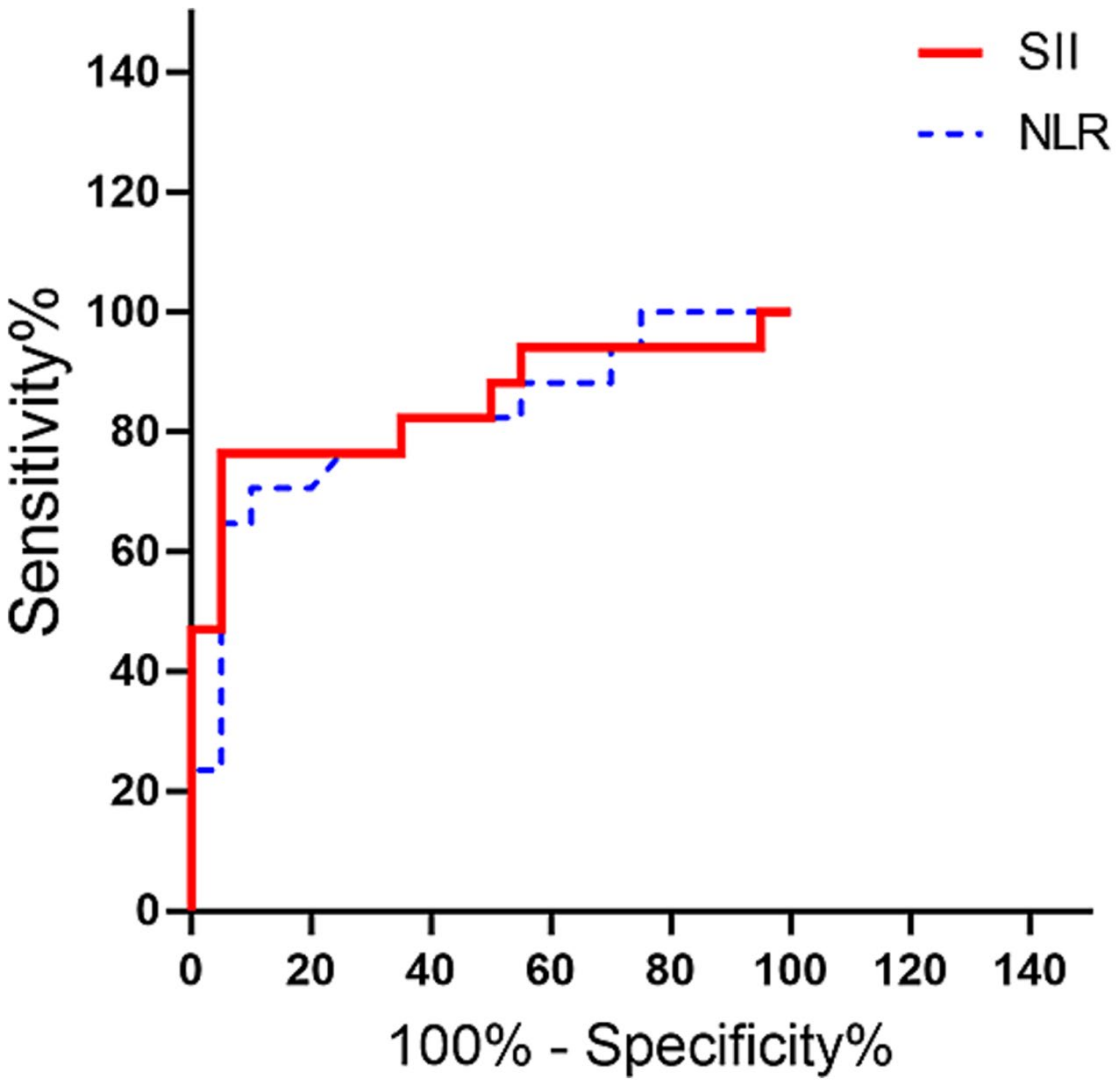
ROC curve analysis of the NLR (blue dotted line) and SII (red line) between the healthy and CVA groups. AUC values were 0.82 (95% CI: 0.68–0.96) for NLR and 0.85 (95% CI: 0.71–0.99) for the SII. The point of intersection on each ROC curve represents the optimal cutoff value (sensitivity and specificity) of 3.92 (70.6% [95% CI: 47.1–88.2%] and 90.0% [95% CI: 75.0–100.0%]) for the NLR and 1,352 (76.5% [95% CI: 52.9–94.1%] and 90.0% [95% CI: 75.0–100.0%]) for the SII. Internal validation was performed using 2,000 bootstrap replicates. AUC, Area Under the Receiver Operating Characteristic curve; CI, confidence interval; CVA, cerebrovascular accident; NLR, neutrophil-to-lymphocyte ratio; ROC, receiver operating characteristic; SII, systemic immune-inflammation index.

For the SII, the AUC was slightly higher at 0.85 (95% CI: 0.71–0.99; *p* < 0.001), with an optimal cutoff value of 1,352, yielding a sensitivity of 76.5% (95% CI: 52.9–94.1%) and specificity of 90.0% (95% CI: 75.0–100.0%). The PPV was 87.5% (95% CI: 71.4–100.0%) and the NPV was 81.8% (95% CI: 69.6–95.0%). The age-adjusted AUC for the NLR improved to 0.86 (95% CI: 0.74–0.99; *p* < 0.001), while the age-adjusted AUC for the SII remained high at 0.85 (95% CI: 0.72–0.99; *p* < 0.001), indicating robust diagnostic accuracy independent of age (Figure 4).

### 3.6 Association between inflammatory markers and clinical outcome

The correlation between the NLR and SII was assessed within each group. In healthy dogs, NLR and SII were strongly correlated (*r* = 0.86; 95% CI: 0.66–0.94; *p* < 0.001). A similarly strong positive correlation was observed in dogs with CVA (*r* = 0.86; 95% CI: 0.64–0.95; *p* < 0.001).

To evaluate the clinical relevance of inflammatory markers, correlations between NLR and SII with CFSS₀ and 30-day survival were assessed. NLR values measured at presentation were not significantly associated with CFSS₀, reflecting the neurological status at the time of admission (*r* = 0.14; 95% CI: −0.38 to 0.59; *p* = 0.598). The SII showed a similarly weak and non-significant correlation with initial clinical severity (*r* = 0.19; 95% CI: −0.34 to 0.62; *p* = 0.471).

**Figure 5:**
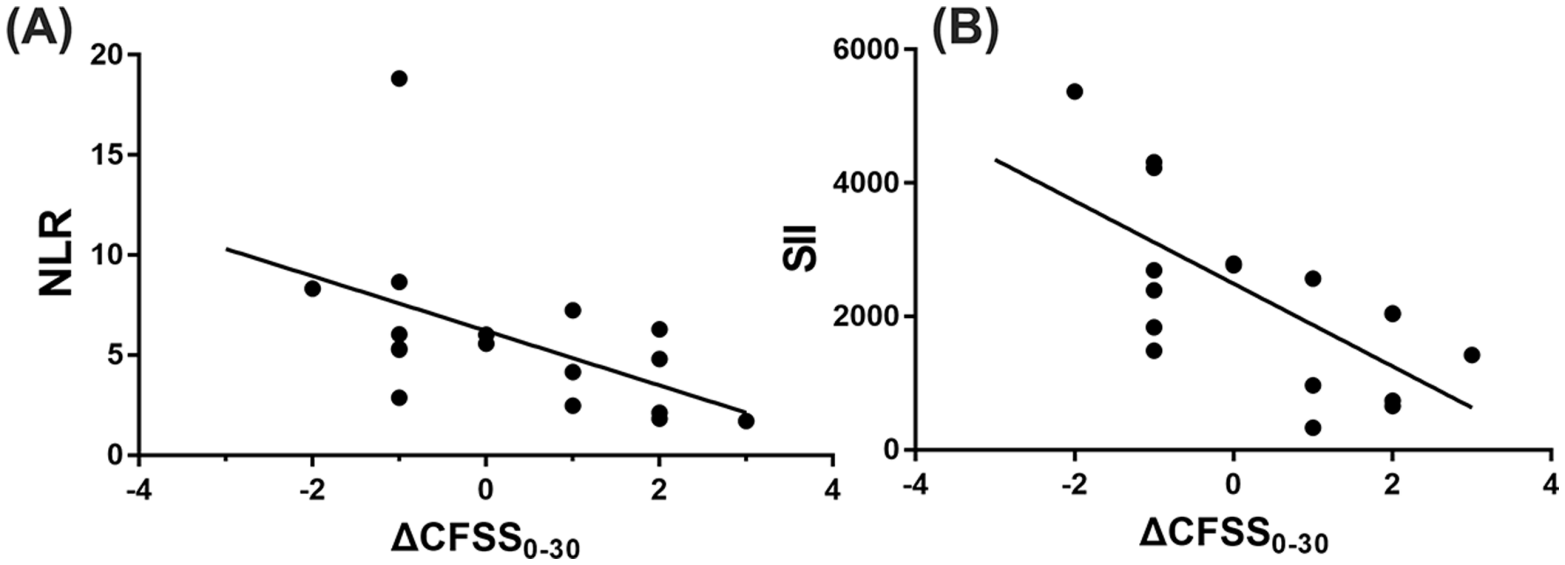
Correlation analysis between **(A)** the NLR and **(B)** SII at presentation and 30-day neurological improvement (ΔCFSS₀_‒_₃₀) in dogs with CVA. The NLR showed a moderate negative correlation with ΔCFSS₀_‒_₃₀ (*r* = −0.60; 95% CI: −0.85 to −0.16; *p* = 0.012) **(A)**, and the SII showed a similarly significant negative correlation (*r* = −0.64; 95% CI: −0.86 to −0.22; *p* = 0.007) **(B)**, indicating that higher inflammatory marker values were associated with poorer functional recovery. A linear regression line with 95% confidence bands is shown. CFSS, Canine Functional Stroke Score; CVA, cerebrovascular accident; NLR, neutrophil-to-lymphocyte ratio; SII, systemic immune-inflammation index.

However, the NLR demonstrated a moderate negative correlation with functional improvement over 30 days, as measured by ΔCFSS₀_‒_₃₀ (*r* = −0.60; 95% CI: −0.85 to −0.16; *p* = 0.012), indicating that dogs with higher NLR at onset tended to have poorer recovery. The SII was also significantly negatively correlated with ΔCFSS₀_‒_₃₀ (*r* = −0.64; 95% CI: −0.86 to −0.22; *p* = 0.007, Figure 5). Among the two markers, the SII demonstrated a slightly stronger and more consistent association with 30-day functional recovery.

To address the potential influence of elective euthanasia on functional improvement scores, a sensitivity analysis was performed by excluding dogs that were euthanized. In this sub-analysis, the negative correlations between inflammatory markers and ΔCFSS₀_‒_₃₀ remained statistically significant for both the NLR (*r* = −0.64; 95% CI: −0.88 to −0.11; *p* = 0.022) and the SII (*r* = −0.63; 95% CI: −0.88 to −0.10; *p* = 0.024).

Regarding 30-day outcomes, seven dogs died during the follow-up period. Four were euthanized and three died from CVA-related complications. No dogs died from causes unrelated to CVA during this period. Detailed descriptions of these cases are provided in Table 4.

**Table 4:** Description of cases.

Case	Breed	Age (year)	Sex	Anatomic location	Type	Final status (day of death)	Comorbidity
1	Spitz	12.6	SF	Midbrain, thalamus	Ischemic, lacunar	Alive	–
2	Maltese	11.0	SF	Thalamus, brainstem, cerebellum	Ischemic, lacunar	Euthanasia (Day 14)	–
3	Poodle	14.0	SF	Cerebrum, cerebellum	Ischemic, territorial	Alive	Lymphangiectasia
4	Maltese	13.3	SF	Cerebellum	Ischemic, lacunar	Alive	–
5	Mixed	9.9	NM	Cerebellum	Ischemic, lacunar	Alive	–
6	Mixed	16.4	M	Midbrain, hypothalamus	Ischemic, lacunar	Euthanasia (Day 8)	MMVD (ACVIM stage B2)
7	Pomeranian	10.0	M	Cerebellum	Ischemic, territorial	Dead (Day8)	–
8	Spitz	13.5	SF	Brainstem, hypothalamus, cerebellum	Ischemic, territorial	Euthanasia (Day 9)	–
9	Maltese	15.2	NM	Cerebellum	Ischemic, territorial	Dead (Day1)	–
10	Poodle	10.1	NM	Midbrain, cerebellum	Hemorrhagic, territorial	Alive	–
11	Yorkshire Terrier	16.0	SF	Cerebellum	Ischemic, territorial	Dead (Day 4)	Hyperadrenocorticism, systemic hypertension
12	Dachshund	14.8	NM	Cerebrum	Mixed	Euthanasia (Day 4)	–
13	Pomeranian	5.0	NM	Cerebrum, cerebellum	Ischemic, territorial	Alive	Recent (2 day) lung lobectomy for lung lobe torsion
14	Poodle	7.9	NM	Midbrain	Ischemic, lacunar	Alive	–
15	Chihuahua	15.0	SF	Cerebellum	Ischemic, territorial	Alive	–
16	Maltese	17.4	SF	Midbrain	Ischemic, lacunar	Alive	MMVD (ACVIM stage B2) pulmonary hypertension, hypothyroidism
17	Maltese	6.9	M	Cerebrum, cerebellum	Ischemic, territorial	Alive	Splenic infarction

M, Male; F, Female; NM, Neutered male; SF, Spayed female; MMVD, Myxomatous mitral valve disease.

Multivariate Cox proportional hazards regression analysis revealed that neither the NLR (HR = 1.026; 95% CI: 0.844–1.248, *p* = 0.796) nor the SII (per 1,000-unit increase; HR = 1.873; 95% CI: 0.965–3.634, *p* = 0.064) was significantly associated with 30-day survival. The proportional hazards assumption for this multivariate model was satisfied, as confirmed by the Schoenfeld residual test (global *p* = 0.197). Although individual predictors did not reach statistical significance and the AIC increased from 34.03 to 35.09, the overall model demonstrated a significant association with 30-day survival (Score log-rank test *p* = 0.04).

In the dichotomized Cox analysis, the SII model (cutoff: 1,352) yielded a further improved AIC of 31.17, although complete separation of outcomes in this cohort resulted in an exceptionally high hazard ratio. In contrast, the dichotomized NLR model (cutoff: 3.92) resulted in an increased AIC of 35.08 (HR = 2.586; 95% CI: 0.4393–48.92; *p* = 0.380), indicating a poorer model fit compared to the continuous model.

**Figure 6:**
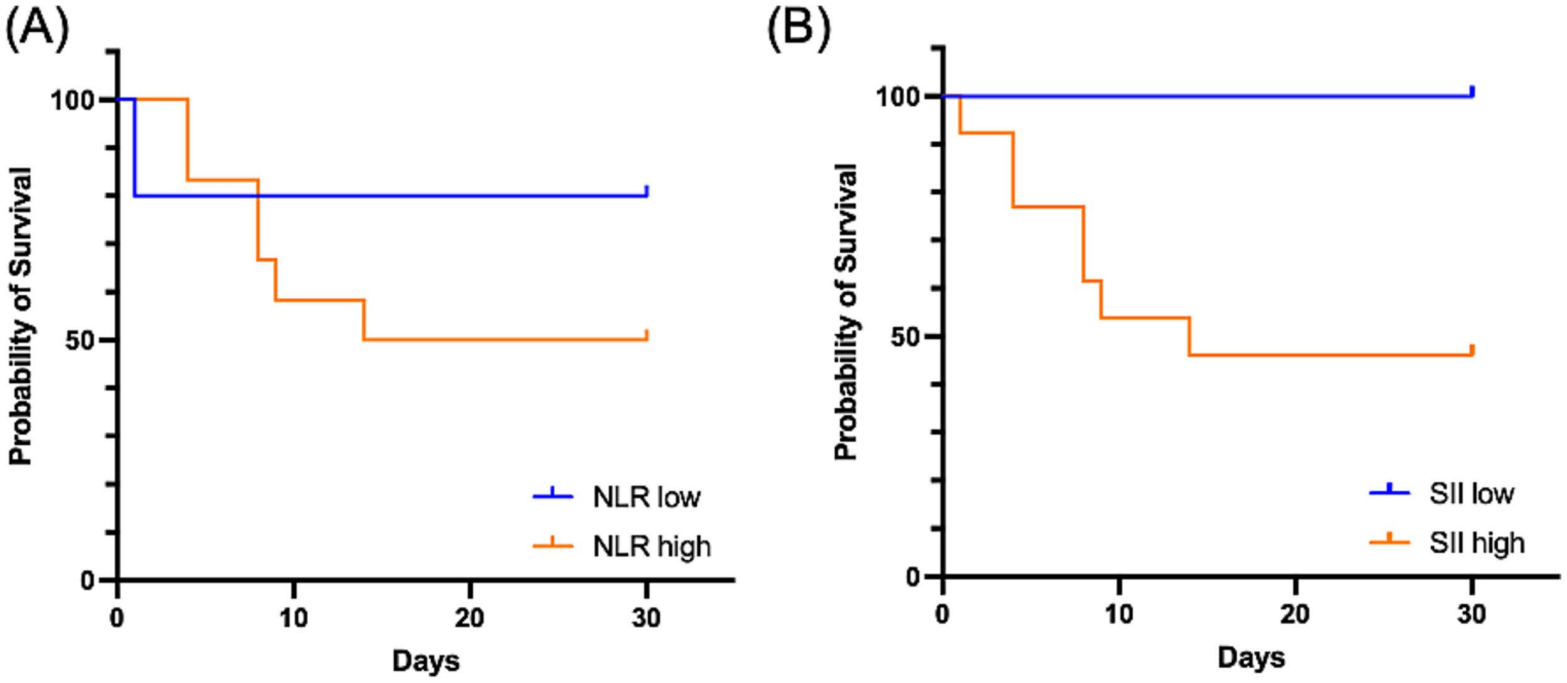
Kaplan–Meier survival curves for 30-day mortality according to optimal cutoffs of the NLR **(A)** and SII **(B)** in dogs with CVA. For the NLR (cutoff: 3.92), no significant difference in survival was observed between the low-NLR (blue line) and high-NLR groups (orange line) (Log-rank *p* = 0.367) **(A)**. For the SII (cutoff: 1,352), the 30-day survival rate was lower in the high-SII group (orange line) compared to the low-SII group (blue line) (Log-rank *p* = 0.086) **(B)**. CI CVA, cerebrovascular accident; NLR, neutrophil-to-lymphocyte ratio; SII, systemic immune-inflammation index.

Kaplan–Meier survival analysis showed that for the SII (cutoff: 1,352), the 30-day survival rate was lower in the high-risk group compared to the low-risk group (Log-rank *p* = 0.086). For the NLR (cutoff: 3.92), no significant difference in survival was observed between the groups (Log-rank *p* = 0.367) (Figure 6).

To further explore group-level differences, inflammatory markers were compared between survivors (≥30 days) and non-survivors. NLR tended to be higher in non-survivors (6.02 [5.30–8.49]) than in survivors (4.48 [2.22–5.92]), although the difference was not statistically significant (*p* = 0.088). In contrast, SII was significantly higher in non-survivors (2767.02 [2115.01–4270.53]) than in survivors (1729.89 [795.16–2438.28]; *p* = 0.043), suggesting that SII has potential utility for predicting short-term mortality in dogs with CVA (Figure 2).

## 4 Discussion

In this study, the diagnostic and prognostic utility of CBC-derived inflammatory markers, specifically the NLR and SII, was assessed in dogs with CVA. Compared to healthy controls, dogs with CVA showed significantly higher NLR and SII values and lower hematocrit, hemoglobin, and red blood cell counts. Both the NLR and SII demonstrated good diagnostic accuracy in differentiating dogs with CVA from healthy dogs, with the SII demonstrating a slightly higher AUC. Although neither marker was significantly associated with 30-day survival in the Cox regression analysis, the overall model showed a significant association with survival. The SII was significantly higher in non-survivors and was negatively correlated with functional improvement over 30 days, suggesting its prognostic relevance. In the dichotomized survival analysis, the high-SII group demonstrated lower survival rates than the low-SII group and an improved model fit for predicting mortality, whereas no significant survival difference was observed between the NLR groups. The CFSS₀ was significantly higher in non-survivors, indicating that greater initial neurological deficits were associated with short-term mortality. However, no significant association was observed between the CFSS₀ and the magnitude of functional improvement, reflecting the variability in recovery trajectories among dogs with CVA.

In our study, higher CFSS₀ scores were significantly associated with 30-day mortality. However, these scores did not consistently predict the degree of functional recovery. This underscores the complex and individualized nature of post-CVA recovery, which may be influenced by factors such as lesion characteristics, comorbidities, and treatment strategies.

Previous studies have explored various systemic blood biomarkers in dogs with CVA, but these markers demonstrate significant diagnostic limitations. For instance, while plasma D-dimer concentrations exhibit high specificity (86.4%), their markedly low sensitivity (30.8%) limits their negative predictive value, meaning a concentration within the reference range cannot reliably exclude a CVA (36). Similarly, C-reactive protein (CRP) and fibrinogen levels often remain within reference limits in dogs with CNS diseases including ischemic CVA and MUO and relevant veterinary investigations remain sparse (37–40). To overcome these diagnostic gaps, we evaluated CBC-derived inflammatory markers, which have shown significant clinical utility in human CVA and other canine CNS diseases (16, 18, 22–24).

The NLR and SII were significantly higher in dogs with CVA than in healthy controls. Among individual cellular components, only the neutrophil count significantly increased, whereas the lymphocyte and platelet counts did not differ significantly. Neutrophils are rapidly recruited to the ischemic brain tissue as part of the acute inflammatory cascade and release cytotoxic mediators, such as reactive oxygen species and pro-inflammatory cytokines, which exacerbate tissue injury and compromise the integrity of the blood–brain barrier (41, 42). In parallel, platelet activation occurs in response to endothelial damage, and platelets interact with neutrophils to form aggregates that further propagate inflammation and thrombosis (43).

Elevated NLR and SII values have been consistently documented in human patients with CVA (44, 45). These markers have also been evaluated for their diagnostic utility (46), supporting their potential as adjunctive tools in clinical neurology. However, dogs with CVA in this study were significantly older than healthy controls. Age-matching was unfeasible due to the limited number of eligible candidates in the healthy control group that met the inclusion criteria. Previous research has shown that both the NLR and SII are moderately correlated with age in healthy dogs (47, 48). Therefore, some of the observed elevations in the NLR and SII in dogs with CVA could reflect age-related hematological changes, potentially leading to an overestimation of these markers in the CVA group. To account for this disparity, we performed an age-adjusted analysis, which demonstrated that the diagnostic performance of these markers remained robust.

The NLR is an inexpensive and readily available index derived from routine CBC, and it reflects the balance between neutrophil-mediated innate immunity and lymphocyte-driven adaptive immunity. It is widely used in both human and veterinary medicine to assess systemic inflammation under various conditions (17–19). The SII, a more recently proposed marker, incorporates platelet counts into the equation by multiplying them with the NLR, thereby integrating prothrombotic and inflammatory components into a single index (20, 21). By reflecting the relative distribution of neutrophils, lymphocytes, and platelets, both the NLR and SII may provide a comprehensive representation of the systemic immune-inflammatory status (49). These indices, calculated from routine hematological parameters, are increasingly recognized as objective measures for assessing disease severity and prognosis (25, 50).

In this study, both the NLR and SII effectively distinguished dogs with CVA from healthy controls, with the SII demonstrating slightly superior discriminatory performance. These findings indicate that CBC-derived inflammatory indices may serve as useful adjuncts for the clinical assessment of suspected CVA. However, because the NLR has also been shown to be elevated in other inflammatory neurological diseases, such as MUO (18), the specificity of these markers for distinguishing CVA from other causes of neuroinflammation may be limited. Therefore, while these indices may assist in identifying the presence of an underlying inflammatory response, they should be interpreted alongside clinical signs and diagnostic imaging findings to ensure an accurate diagnosis.

In addition to diagnostic performance, the associations of the NLR and SII with clinical outcomes, including functional recovery and short-term survival, were investigated. Interestingly, neither NLR nor SII correlated with initial neurological severity at presentation, suggesting that systemic inflammation at the time of diagnosis does not directly reflect baseline clinical status. This may be explained by several factors unique to veterinary clinical settings. In dogs, the exact onset time of cerebrovascular events is often uncertain, and delays in presentation may result in a temporal mismatch between the systemic inflammatory status and neurological examination findings (51). To mitigate this potential mismatch, we limited our inclusion to dogs presenting within 3 days (72 h) of symptom onset. This timeframe was established based on evidence from human medicine indicating that inflammatory markers such as the NLR typically peak at 24 h and begin to decline at 72 h following an ischemic CVA (52). Furthermore, infarcts in specific brain regions may cause substantial neurological impairment with only modest systemic inflammation, thereby attenuating the association between these markers and initial clinical severity (53).

However, both markers demonstrated a moderate and statistically significant negative correlation with ΔCFSS₀_‒_₃₀, representing functional improvement over 30 days. Higher NLR and SII values at presentation were associated with smaller reductions in CFSS, indicating poorer functional recovery. To account for potential bias from elective euthanasia, a sensitivity analysis was performed excluding these cases. The correlations remained statistically significant, confirming the robustness of the results. These findings suggest that systemic inflammation influences the course of recovery in dogs with CVA and highlight the potential prognostic value of the NLR and SII in this context.

Comparable associations have been reported in human patients with CVA. An elevated NLR at admission has been correlated with greater CVA severity and worse functional outcomes in multiple clinical studies and meta-analyses (16, 44). Similarly, increased SII values have been associated with more severe neurological deficits and unfavorable outcomes after acute ischemic CVA, particularly in patients undergoing thrombolytic therapy (22, 45). These similarities reinforce the relevance of CBC-derived inflammatory indices for predicting post-CVA prognosis across species.

To further assess the prognostic utility of systemic inflammatory markers, we analyzed their association with 30-day survival. In the multivariate Cox proportional hazards regression model, neither the NLR nor the SII were significantly associated with survival. Although individual predictors did not reach statistical significance, the overall model demonstrated a significant association with 30-day survival, suggesting a collective prognostic value when these markers are combined. The prognostic value of these markers became more apparent through dichotomized analysis. The dichotomized SII model demonstrated a more robust fit than the continuous model, suggesting that the risk associated with SII may not follow a linear pattern but rather increases beyond a specific threshold. Similarly, in the Kaplan–Meier analysis, the high-SII group exhibited a lower 30-day survival rate, though this difference was not statistically significant. In contrast, dichotomizing the NLR resulted in a poorer model fit, indicating that the NLR may have less predictive power for mortality. When survival status was assessed by direct group-wise comparison, SII values at presentation were significantly higher in non-survivors than in survivors, whereas the difference in NLR values did not reach significance. These findings suggest that an elevated SII may be associated with short-term mortality risk in dogs with CVA and support its potential utility as a prognostic marker. It is worth noting that while the SII showed a significant difference in a direct comparison between survivors and non-survivors, it did not reach statistical significance in the Cox proportional hazards model. This discrepancy suggests that while higher SII levels are associated with the overall occurrence of 30-day mortality, they may not be a significant independent predictor of the specific timing of death within that period. Nevertheless, the group-wise difference reflects a general trend where elevated systemic inflammation is present in dogs with poor clinical outcomes, even if it lacks the temporal precision provided by the Cox model.

In this study, the SII demonstrated stronger and more consistent associations than the NLR across both diagnostic performance and clinical outcome measures. These findings indicate that the SII may better reflect the combined inflammatory and thrombotic processes underlying acute cerebrovascular injury in dogs. The prognostic superiority of the SII over the NLR has also been observed in CVA research in humans. Unlike the NLR, the SII incorporates platelet count, enhancing its sensitivity to thromboinflammatory activity following cerebrovascular events (24). Activated platelets play a critical role in CVA pathophysiology by adhering to damaged endothelium, initiating inflammatory cascades, and promoting thrombus formation through the release of bioactive mediators such as serotonin, platelet-derived growth factor, and transforming growth factor-β (54, 55). Through these mechanisms, platelet activation contributes to vascular injury and impairs neurological recovery.

The CFSS was developed as a pragmatic, semi-quantitative measure to approximate acute functional severity, conceptually adapted from human scales such as the mRS and NIHSS (26, 27). It primarily emphasizes ambulation and mentation, as these domains serve as reliable indicators of global disability. However, we acknowledge that several qualitative descriptors within the scale are inherently subjective and may introduce inter-observer variability. Furthermore, the CFSS does not fully capture certain focal deficits, such as visual impairment, circling, or vestibular signs, which may not directly correlate with ambulatory status. Despite these limitations, a function-oriented approach was considered appropriate because the primary aim of this study was to evaluate the association between systemic inflammatory markers and overall functional recovery rather than specific neurological syndromes. Consequently, the CFSS should be interpreted as an exploratory functional metric requiring further validation to enhance its discriminatory power.

Several comorbidities were identified in dogs with CVA in our cohort, including myxomatous mitral valve disease (MMVD) American College of Veterinary Internal Medicine (ACVIM) stage B2 (56), pulmonary hypertension, hypothyroidism, and hyperadrenocorticism (HAC). Most dogs were medically managed prior to the onset of CVA. However, these comorbidities may have influenced baseline NLR and SII and potentially affected clinical outcomes. The impact of comorbidities on inflammatory indices appears to be disease specific. A recent study in dogs with MMVD reported that total WBC counts and NLR were not significant predictors of survival (57), whereas HAC has been associated with significant alterations in both NLR and platelet to lymphocyte ratio (PLR), serving as markers for hypercortisolism (58). Given that the effects of many chronic diseases on complex indices like NLR and SII remain largely unexplored in veterinary medicine, our findings should be interpreted with these potential baseline elevations in mind. Comorbidities may also have contributed to a hypercoagulable state leading to CVA. Conditions such as hyperadrenocorticism, systemic hypertension, hypothyroidism, and lymphangiectasia may have predisposed dogs to a hypercoagulable state and contributed to subsequent cerebrovascular events (59). One dog underwent lung lobectomy for lung lobe torsion shortly before the CVA. Post-surgical inflammation and the underlying pathology could have elevated NLR and SII and potentially precipitated the cerebrovascular event. Regarding survival, none of the dogs died directly from their comorbidities, but their potential impact should still be considered. A recent study found that dogs with comorbidities tended to have shorter post-CVA survival, although this difference did not reach statistical significance (59).

This study had several limitations. First, this was a retrospective, single-center study with a small sample size, limiting the statistical power and generalizability of the findings. Specifically, the small number of hemorrhagic cases precluded a meaningful subtype-specific analysis, as inflammatory profiles may differ between ischemic and hemorrhagic CVA, and our findings may not fully capture these distinctions. Second, while the CFSS was developed to address the specific needs of canine CVA assessment, it lacks formal validation in veterinary neurology and remains to be externally validated. Furthermore, because the assessment of neurological status using the CFSS was conducted by the primary attending clinician for each case, a formal inter-rater reliability assessment was unfeasible. While this lack of calibration represents a significant limitation and introduces potential subjective bias, it is important to note that the core components of the CFSS—such as ambulation status and presence of deep pain perception—represent objective clinical milestones that are less susceptible to major inter-observer variance. By having the same clinician perform both baseline and follow-up assessments for each patient, we also aimed to ensure internal consistency in measuring the magnitude of functional recovery (∆CFSS). Nevertheless, the current results should be interpreted as a preliminary functional exploration. Third, the timing of clinical presentation and blood sampling could not be standardized, potentially leading to discrepancies between the inflammatory marker levels and clinical severity. Moreover, the NLR and SII are non-specific indices that may be influenced by various unmeasured confounders, including comorbidities, concurrent medications, or systemic stress responses. In addition, drugs such as anti-inflammatory agents, antiplatelet therapies, or corticosteroids can substantially affect inflammation levels and platelet counts, thereby altering NLR and SII values. Due to the retrospective nature of this study, the effects of medications administered for the management of comorbidities could not be fully controlled or excluded. Finally, the medical management was not standardized across cases, and treatment variability may have affected the clinical outcomes.

Despite these limitations, this study provides preliminary insights into the relationship between systemic inflammation and clinical outcomes of canine CVA. In the future, NLR and SII, which are readily obtainable from routine complete blood counts, may provide supportive information in dogs with suspected CVA. These markers could help predict clinical outcome, elucidate the influence of systemic inflammation on disease progression, and potentially assist in guiding therapeutic decisions. To date, investigations of inflammatory markers in this context are scarce. Our findings contribute to a growing understanding of CVA pathophysiology in veterinary neurology. Future prospective multicenter studies with larger cohorts and standardized protocols are warranted to validate these findings and further define the prognostic utility of CBC-derived inflammatory indices in dogs with cerebrovascular disease.

## 5 Conclusion

In this study, we evaluated the diagnostic and prognostic utility of NLR and SII in dogs with CVA. Both markers were significantly elevated in affected dogs compared to those in healthy controls and were associated with the degree of functional recovery. Notably, the SII also demonstrated a significant association with short-term survival, suggesting its potential value as a prognostic indicator. These findings support the use of CBC-derived inflammatory indices as accessible adjuncts in the clinical evaluation of canine CVA and provide preliminary evidence linking systemic inflammation with post-CVA outcomes.

## Data Availability

The original contributions presented in the study are included in the article/Supplementary material, further inquiries can be directed to the corresponding authors.
