# Supplementary material for: Complete blood count-derived inflammatory markers in canine cerebrovascular accidents: diagnostic utility and prognostic implications
Source: Front Vet Sci. 2026 Apr 22;13:1719067. doi: 10.3389/fvets.2026.1719067 (PMC13144770; doi:10.3389/fvets.2026.1719067)
Supplement: Supplementary file 1 [file Table_1.docx]

**Supplementary Table S1.** Detailed description of magnetic resonance imaging findings and vascular territories for individual cases.

| **Case** | **Anatomic Location** | **Type** | **Vascular Territory** | **ADC/DWI Availability** | **Contrast Enhancement** | **Artifact** |
| --- | --- | --- | --- | --- | --- | --- |
| 1 | Midbrain, thalamus | Ischemic, lacunar | Lt. Paramedian Perf AA | Available | Not enhanced | None significant |
| 2 | Thalamus, brainstem, cerebellum | Ischemic, lacunar | Multifocal Perf AA, bilateral RosCA | Available | Not enhanced | None significant |
| 3 | Cerebrum, cerebellum | Ischemic, territorial | Lt. PCA, bilateral RosCA | Available | Not enhanced | None significant |
| 4 | Cerebellum | Ischemic, lacunar | RosCA | Available | Not enhanced | None significant |
| 5 | Cerebellum | Ischemic, lacunar | Lt. RosCA, CauCA | Available | Not enhanced | None significant |
| 6 | Midbrain, hypothalamus | Ischemic, lacunar | Lt. Paramedian Perf AA | Available | Not enhanced | None significant |
| 7 | Cerebellum | Ischemic, territorial | Rt. RosCA | Available | Not enhanced | None significant |
| 8 | Brainstem, hypothalamus, cerebellum | Ischemic, territorial | Lt. RosCA | Available | Not enhanced | None significant |
| 9 | Cerebellum | Ischemic, territorial | Rt. RosCA | Available | Not enhanced | None significant |
| 10 | Midbrain, cerebellum | Hemorrhagic, territorial | Lt. RosCA | Available | Not enhanced | None significant |
| 11 | Cerebellum | Ischemic, territorial | Lt. RosCA | Available | Not enhanced | None significant |
| 12 | Cerebrum | Mixed | Rt. MCA | Available | Grey matter, leptomeningeal enhancement | None significant |
| 13 | Cerebrum, cerebellum | Ischemic, territorial | Bilateral PCA, Lt. RosCA | Available | Not enhanced | None significant |
| 14 | Midbrain | Ischemic, lacunar | Lt. Paramedian Perf AA | Available | Not enhanced | None significant |
| 15 | Cerebellum | Ischemic, territorial | Rt. RosCA | Available | Not enhanced | None significant |
| 16 | Midbrain | Ischemic, lacunar | Rt. Paramedian Perf AA | Available | Not enhanced | None significant |
| 17 | Cerebrum, cerebellum | Ischemic, territorial | Bilateral MCA, RosCA | Available | Not enhanced | None significant |

**Note:** Lt, Left; Rt, Right; MCA, Middle Cerebral Artery; PCA, Posterior Cerebral Artery; RosCA, Rostral Cerebellar Artery; CauCA, Caudal Cerebellar Artery; Perf AA, Perforating Arteries; ADC, Apparent diffusion coefficient; DWI, Diffusion-weighted imaging.
